# Network pharmacology and molecular docking analysis on molecular targets and mechanisms of scar healing ointment in the treatment of hypertrophic scars

**DOI:** 10.3389/fphar.2025.1511570

**Published:** 2025-08-06

**Authors:** Lijuan Jiang, Xiandong Zeng, Hongjun Li, Jingping Wu

**Affiliations:** ^1^ Department of Medical College, Chengdu University of Traditional Chinese Medicine, Chengdu, Sichuan, China; ^2^ Department of Medical Cosmetology, Chengdu University of Traditional Chinese Medicine, Chengdu, Sichuan, China

**Keywords:** hypertrophic scars, traditional Chinese medicine, scar healing ointment, network pharmacology, molecular docking

## Abstract

**Introduction:**

Hypertrophic scars (HSs) are characterized by complex mechanisms and impose substantial economic and psychological burdens on patients with wounds. Recent studies have reported that various extracts from traditional Chinese medicines can help prevent and treat HSs. Scar healing ointment (SHO), a modified traditional Chinese prescription applied externally, has demonstrated potential in the clinical treatment of HSs, though its underlying mechanisms remain unexplored.

**Methods:**

In this study, we systematically identified the active ingredients of the SHO formula and their potential targets using multiple databases (TCMSP, HERB, UniProt, GeneCards, DisGeNet, OMIM, PharmGKB, TTD) and explored the possible underlying mechanisms by which SHO treats HSs using bioinformatic analyses, including protein-protein interaction (PPI) network analysis, GO and KEGG enrichment analyses, and molecular docking.

**Results:**

Our results indicated that the primary active ingredients in the SHO formula include quercetin, beta-sitosterol, kaempferol, stigmasterol, luteolin, alloimperatorin, acacetin, and (E)-2,3-bis(7-methoxy-2-oxochromen-8-yl)prop-2-enal. Protein-protein interaction network analysis revealed that the hub target proteins of the SHO formula are AKT1, MAPK1, CCND1, TP53, GSK3B, BCL2, CDKN1A, ESR1, and MYC. GO and KEGG enrichment analyses showed that these hub target genes are involved in processes and pathways related to apoptosis and responses to oxidants. Molecular docking analysis demonstrated that the MAPK1-stigmasterol and ESR1-alloimperatorin complexes exhibited strong binding affinities (–5.31 and –6.09) and formed multiple hydrogen bonds (3 and 2, respectively).

**Discussion:**

These findings suggest that SHO may exert its effects by modulating MAPK1 and ESR1 proteins, thereby contributing to the prevention and treatment of HSs. This study offers new drugs and target candidates for the prevention and treatment of HSs and provides theoretical support for further research and application of the SHO formula. Nevertheless, additional *in vivo* and *in vitro* studies are necessary to validate these mechanisms.

## Introduction

The skin is the largest organ in the human body and is highly susceptible to physical injury such as surgeries, traumas, and burns (Coentro et al., 2019). To maintain life, the skin must heal quickly, involving a complex physiologic process where the body attempts to replace injured skin with newly generated tissue and restore its barrier functions (Mony et al., 2023). This process involves four stages: hemostasis, inflammation, proliferation, and remodeling, as well as communication between many different types of cells (Gurtner et al., 2008). Under normal conditions, these stages occur in a highly sequential and finite manner, and the myofibroblasts undergo apoptosis during the remodeling stage, leaving a relatively acellular scar at the healed sites (Bharadia et al., 2023). However, this process sometimes does not occur sequentially and finitely, resulting in aberrant wound-healing processes and subsequent fibroproliferative disorders and hypertrophic scars (HSs) (Gurtner et al., 2008; Limandjaja et al., 2021). HSs are characterized by excessive extracellular matrix (ECM), especially collagen, deposition during the wound healing process, leading to raised, often red, thickened scars that remain within the boundaries of the original wound (Diegelmann and Evans, 2004; Lingzh et al., 2020). These scars can be painful and itchy, symptoms that may worsen with elevated ambient temperature, emotional arousal, or consumption of spicy foods (Bharadia et al., 2023; Jeschke et al., 2023), affecting 30%–90% of patients with wounds (Bharadia et al., 2023; Limandjaja et al., 2021) and posing serious social, functional, and economic burdens due to their appearance, contractures, and the costs associated with scar treatment (Thombs et al., 2008; Leblebici et al., 2006; Mcphail et al., 2022). Considering the significant portion of the population suffering from HSs, effectively preventing the formation of HSs during wound healing or removing already formed HSs is of great importance.

Currently, there are two mature clinical measures for preventing and treating the formation of HSs. The first is known as first-line therapy, represented by silicone gel sheeting (Bharadia et al., 2023). For example, silicone gel has been reported to reduce the expression of transforming growth factor (TGF)-β1, platelet-derived growth factor, and basic fibroblast growth factor (b-FGF) 4 months after surgery for surgical scars (Choi et al., 2015). However, other studies have reported that silicone gel can also upregulate the expression of b-FGF in the dermis of silicone gel sheet-treated scars (O’brien and Jones, 2013). The second measure is known as second-line therapy, which includes laser therapy as well as surgical excision in combination with intralesional corticosteroid injections postoperatively (Bharadia et al., 2023; Atiyeh, 2020). For instance, it has been reported that long-pulsed Nd:YAG laser and pulsed dye can lighten the color, reduce the thickness and tension of HSs, and relieve symptoms such as pain and pruritus (Amini-Nik et al., 2018).

In recent years, traditional Chinese medicine (TCM), including various extracts, has been proven effective as a potential therapy for treating HSs due to its roles in promoting skin regeneration and resisting skin aging. For example, many traditional plant-based products, such as quercetin, onion extract, resveratrol, epigallocatechin gallate, oleanolic acid, and curcumin, have been used clinically, providing relief to patients to varying extents (Bharadia et al., 2023). Scar healing ointment (SHO) is a TCM prescription for the external treatment of scarring. It includes ingredients like Wubeizi (*Galla Chinensis*), Wumei (*Mume Fructus*), Baizhi (*Angelicae Dahuricae*), Shechuangzi (*Fructus Cnidii*), Qianliguang (*Senecio Scandens*), and Digupi (*Cortex Lycii*), and has been clinically effective in preventing the formation of HSs and removing already formed HSs. However, the underlying mechanisms of its effects on HS prevention and treatment remain unclear.

Network pharmacology is a promising approach to reveal the pharmacological mechanisms of Chinese medicine formulas (Zhao et al., 2023) and predict their potential targets for specific diseases (Zhang et al., 2017; Kou and Ma, 2024). Molecular docking is a significant method in structural molecular biology and computer-aided drug design (Saikia and Bordoloi, 2019). Therefore, in the present study, we aim to analyze the active ingredients and molecular targets of SHO using network pharmacology and molecular docking methods to explore its potential underlying mechanisms for the prevention and treatment of HSs. Our study will provide new drug and target options for the prevention and treatment of HSs, and offer theoretical support for further research and promotion of SHO.

## Materials and methods

### Identification and screening of active ingredients and their protein targets

The Traditional Chinese Medicine Systems Pharmacology Database and Analysis Platform (TCMSP, https://tcmsp.91medicine.cn/) (Ru et al., 2014) was used to identify all ingredients in the six herbs comprising the SHO formula, namely, Wubeizi, Wumei, Baizhi, Shechuangzi, Digupi, and Qianliguang. The names of these herbs were used as keywords to retrieve all components. As a result, the ingredients of Wubeizi, Wumei, Baizhi, Shechuangzi, and Digupi were obtained from the TCMSP database. However, the keyword “Qianliguang” was not available in TCMSP, so its ingredients were retrieved from the HERB database (http://herb.ac.cn/) (Fang et al., 2021). To ensure consistency, we cross-referenced the chemical abstracts service (CAS) registry number of the ingredients with a list in both databases, and only ingredients present in the TCMSP database were included as active ingredients of Qianliguang. Since the TCMSP database does not provide dermal permeability parameters for the ingredients, we screened these components using the pharmacokinetic parameters oral bioavailability (OB) ≥ 30% and drug-likeness (DL) ≥ 0.18, in accordance with previous studies (Fan et al., 2024; Han et al., 2022), to facilitate subsequent analysis. Afterward, the protein targets associated with the screened active ingredients were retrieved from the TCMSP database, and the gene names of these targets were further extracted using the UniProt database (https://www.uniprot.org/) (UniProt, 2021). To reflect the complex relationships between active ingredients and their potential target genes, we employed the Cytoscape software (v3.9.1; http://www.cytoscape.org/) (Shannon et al., 2003) to establish a visual herb-ingredient-target gene network.

### Predicting the targets of HSs and protein-protein interactions (PPI) analysis

We gathered information on HSs-associated gene targets from the GeneCards database (https://www.genecards.org/), Disease-Gene Network database (DisGeNet; https://www.disgenet.com/), Online Mendelian Inheritance in Man database (OMIM; https://www.omim.org/), Pharmacogenomics Knowledgebase (PharmGKB; https://www.pharmgkb.org/), and Therapeutic Target Database (TTD, https://db.idrblab.org/ttd/) using “hypertrophic scars” as the keyword. The shared gene targets between the HSs-associated gene targets and SHO-associated gene targets were identified as the potential targets of SHO for HSs. These potential gene targets were put into STRING (v12.0; https://string-db.org/cgi/input.pl) to construct the PPI network interaction (interaction score ≥0.900) referencing previous research (Szklarczyk et al., 2019; Islam et al., 2018), which was visualized using Cytoscape software. The classical degree value was used as the standard to measure the importance of nodes in the PPI network, and the CytoNCA plug-in (Tang et al., 2015) in the Cytoscape software was used to calculate a series of degree values. The median degree value calculated from the CytoNCA plug-in, rounded to the nearest integer, was used as the threshold for screening hub genes, referencing previous studies (Fan et al., 2024; Han et al., 2022).

### GO and KEGG enrichment analyses

The Gene Ontology (GO) and Kyoto Encyclopedia of Genes and Genomes (KEGG; https://www.kegg.jp/) enrichment analyses of these SHO-related potential targets were performed using the “ClusterProfiler” package (v 4.10.1) in the RStudio software (v 2023.12.1). GO terms and KEGG pathways with adjusted p-value <0.05 were considered to be significantly enriched.

### Ingredient-target molecular docking

The three-dimensional (3D) structures of the core target proteins were downloaded from the Research Collaboratory for Structural Bioinformatics Protein Data Bank database (PDB; https://www.rcsb.org/). AutoDock Tools software (v1.5.7; https://autodocksuite.scripps.edu/adt/) was then used to process these structures by removing water molecules, isolating proteins, adding nonpolar hydrogen atoms, and calculating Gasteiger charges. The processed structures were saved as PDBQT files to serve as receptors. The 2-dimensional (2D) structures of selected ingredients were downloaded from the PubChem database (https://pubchem.ncbi.nlm.nih.gov/). These 2D structures were then converted into PDB format and saved as docking ligands using the Chem 3D software (v22.0.0.22). The active site for molecular docking was determined based on the ligand coordinates in the target protein complex. The ligand was set to be flexible, while the receptor remained rigid. AutoDock Vina (v1.1.2) was used to dock these ligands with the receptors. For the docking results, multiple conformations were generated, and the conformation with the best affinity was selected as the final docking conformation. Conformations with affinity <−5 were then visualized using Pymol (v 2.3) software.

## Results

### Active ingredients and potential target genes of SHO

From the six herbs, a total of 386 active ingredients were retrieved from the TCMSP database: three in Wubeizi, 43 in Wumei, 114 in Shechuangzi, 222 in Baizhi, 20 in Qianliguang, and 38 in Digupi. After screening for OB ≥ 30% and DL ≥ 0.18, a total of 57 active ingredients were obtained: one in Wubeizi, eight in Wumei, 19 in Shechuangzi, eight in Qianliguang, 13 in Digupi, and 22 in Baizhi (Table 1). Detailed information about the retrieved active ingredients in the six herbs is shown in Supplementary Table S1. Then, a total of 145 target genes associated with these active ingredients were further retrieved from the TCMSP database (Supplementary Table S2).

**TABLE 1:** Active ingredients with oral bioavailability ≥30% and drug-likeness ≥0.18

Drug	Mol ID	Molecule name	OB (%)	DL
Wumei, Shechuangzi, Baizhi, Digupi	MOL000449	Stigmasterol	43.82	0.75
Wumei, Shechuangzi, Baizhi, Digupi	MOL000358	Beta-sitosterol	36.91	0.75
Wumei	MOL005043	Campest-5-en-3beta-ol	37.57	0.71
Wumei, Baizhi, Digupi	MOL000953	CLR	37.87	0.67
Wumei, Qianliguang	MOL000098	Quercetin	46.43	0.27
Wumei, Qianliguang	MOL000422	Kaempferol	41.88	0.24
Wumei	MOL008601	Methyl arachidonate	46.89	0.23
Wumei	MOL001040	(2R)-5,7-dihydroxy-2-(4-hydroxyphenyl)chroman-4-one	42.36	0.21
Wubeizi	MOL000569	Digallate	61.85	0.26
Shechuangzi	MOL001771	Poriferast-5-en-3beta-ol	36.91	0.75
Shechuangzi	MOL003605	(E)-2,3-bis(2-keto-7-methoxy-chromen-8-yl)acrolein	56.37	0.71
Shechuangzi	MOL001510	24-epicampesterol	37.57	0.71
Shechuangzi	MOL003591	Ar-curcumene	52.34	0.64
Shechuangzi	MOL003607	Cniforin B	36.69	0.6
Shechuangzi	MOL003606	Cniforin A	55.88	0.47
Shechuangzi	MOL003624	O-Isovalerylcolum bianetin	64.02	0.35
Shechuangzi	MOL003604	Cnidimol F	54.43	0.28
Shechuangzi	MOL002881	Diosmetin	31.13	0.27
Shechuangzi	MOL003600	Cnidimol B	68.65	0.25
Shechuangzi	MOL003608	O-Acetylcolumbianetin	60.03	0.25
Shechuangzi	MOL003617	Isogosferol	30.07	0.25
Shechuangzi	MOL003626	Ostruthin	30.64	0.23
Shechuangzi, Baizhi	MOL001941	Ammidin	34.54	0.22
Shechuangzi, Baizhi	MOL003588	Prangenidin	36.31	0.21
Shechuangzi	MOL003584	Xanthoxylin N	35.5	0.2
Shechuangzi, Baizhi	MOL002883	Ethyl oleate (NF)	32.39	0.19
Qianliguang	MOL000665	Flemiphilippinin C	47.66	0.73
Qianliguang, Digupi	MOL001790	Linarin	39.84	0.7
Qianliguang	MOL004492	Chrysanthemaxanthin	38.72	0.58
Qianliguang	MOL002680	Flavoxanthin	60.41	0.55
Qianliguang	MOL010023	Senkirkine	56.15	0.4
Qianliguang	MOL000006	Luteolin	36.16	0.24
Digupi	MOL002228	Kulactone	45.43	0.81
Digupi	MOL000296	Hederagenin	36.91	0.75
Digupi	MOL002224	Aurantiamide acetate	58.38	0.58
Digupi	MOL002218	Scopolin	56.44	0.38
Digupi	MOL002222	Sugiol	36.11	0.27
Digupi	MOL001689	Acacetin	34.97	0.24
Digupi	MOL002219	Atropine	34.52	0.21
Digupi	MOL001552	OIN	45.97	0.19
Digupi	MOL001645	Linoleyl acetate	42.1	0.19
Baizhi	MOL005807	Sen-byakangelicol	58	0.61
Baizhi	MOL001506	Supraene	33.54	0.42
Baizhi	MOL005800	Byakangelicol	41.42	0.35
Baizhi	MOL001749	ZINC03860434	43.59	0.34
Baizhi	MOL005789	Neobyakangelico l	36.18	0.31
Baizhi	MOL013430	Prangenin	43.59	0.29
Baizhi	MOL005806	4-[(2S)-2,3-dihydroxy-3-methylbutoxy]furo [3,2-g]chromen-7-one	39.98	0.29
Baizhi	MOL003791	Linolein, 2-mono-	37.28	0.29
Baizhi	MOL002644	Phellopterin	40.18	0.27
Baizhi	MOL001956	Cnidilin	32.68	0.27
Baizhi	MOL005792	5-[2′(R)-Hydroxy-3′-methyl-3′-butenyl-oxy]furocoumarin	42.85	0.25
Baizhi	MOL005802	propyleneglycol monoleate	37.6	0.25
Baizhi	MOL001942	Isoimperatorin	45.46	0.22
Baizhi	MOL007514	methyl icosa-11,14-dienoate	39.66	0.22
Baizhi	MOL001939	Alloisoimperatorin	34.8	0.21
Baizhi	MOL001494	Mandenol	41.99	0.19

Note: OB, oral bioavailability; DL, Drug-likeness.

### HSs associated genes and potential target genes of SHO for HSs

**FIGURE 1:**
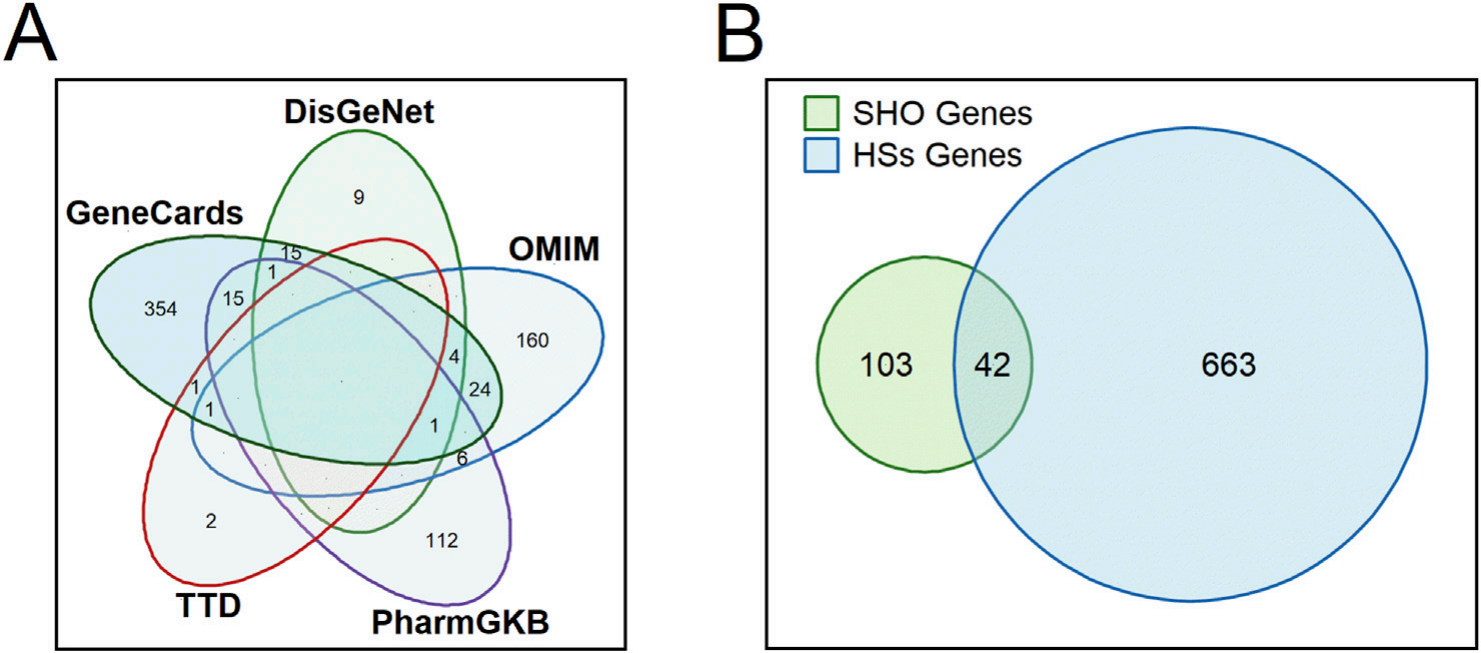
Venn diagrams showing the number of retrieved hypertrophic scars-associated genes from each database **(A)** and shared genes between the 145 target genes associated with the 57 active ingredients and the 705 genes associated with HSs. SHO, scar healing ointment; HSs, hypertrophic scars. **(B)** displays a Venn diagram of SHO genes and HSs genes, with overlapping regions labeled to indicate the number of shared genes (n = 42), where SHO refers to scar healing ointment and HSs refers to hypertrophic scars.

We retrieved a total of 705 genes associated with HSs from five databases: 30 from the DisGeNet database, 197 from the OMIM database, 135 from the PharmGKB database, four from the TTD database, and 416 from the GeneCards database (Figure 1A). Detailed information about these HSs-associated genes is provided in Supplementary Table S3. Subsequently, it was identified that 42 core genes were shared between the 145 target genes associated with the 57 active ingredients and the 705 genes associated with HSs (Figure 1B). Supplementary Table S4 provides detailed information about the shared and unique genes between these active ingredients-associated genes and HSs-associated genes.

### Herb-ingredient-target gene network

**FIGURE 2:**
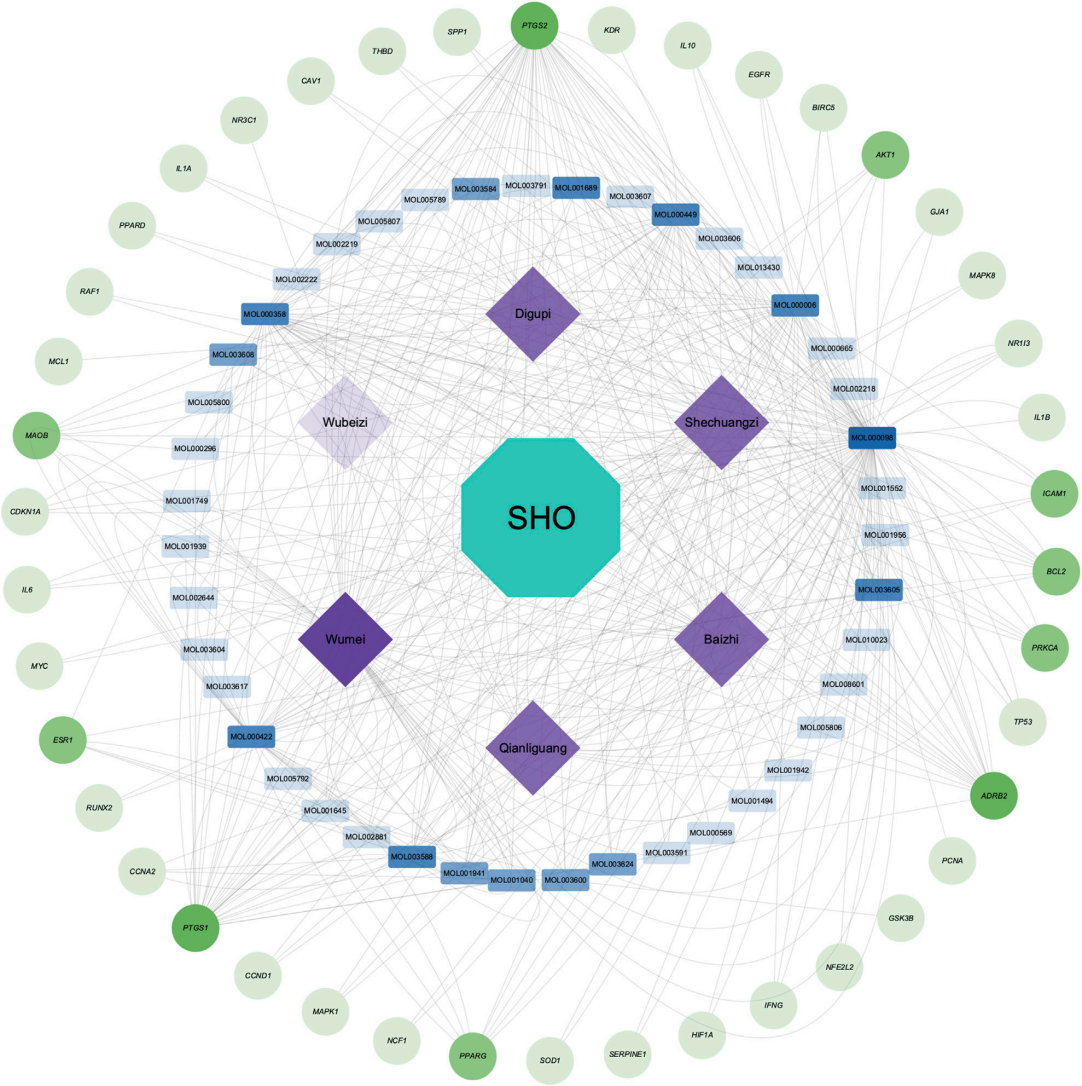
Herb-ingredient-target gene network of scar healing ointment (SHO). The green circle nodes in the outmost circle represent the 42 target genes, the blue rectangular nodes in the inner circle represent the 43 active ingredients with target genes, and the rhombic purple nodes in the innermost circle represent the six herbal medicines in the SHO. The transparency of the nodes reflects their Degree values: the lower transparency indicates higher Degree values.

After removing 14 active ingredients without target genes, an herb-ingredient-target gene network was constructed. This network contains 91 nodes, including six herbs, 43 active ingredients, and 42 target genes, as well as 442 edges (Figure 2). Based on their degree values (≥10), we considered Mol000098, Mol000358, Mol000422, Mol000449, Mol000006, Mol003588, Mol001689, and Mol003605 as the core active ingredients in SHO. More detailed information about these active ingredients is provided in Table 2.

**TABLE 2:** Detailed information about the core active ingredients in scar healing ointment.

Mol ID	Degree value	Ingredient	PubChem ID	Molecular formula
Mol000098	140	Quercetin	5280343	C_15_H_10_O_7_
Mol000358	40	Beta-Sitosterol	222284	C_29_H_50_O
Mol000422	36	Kaempferol	5280863	C_15_H_10_O_6_
Mol000449	32	Stigmasterol	5280794	C_29_H_48_O
Mol000006	32	Luteolin	5280445	C_15_H_10_O_6_
Mol003588	16	Alloimperatorin	69502	C_16_H_14_O_4_
Mol001689	12	Acacetin	5280442	C_16_H_2_O_5_
Mol003605	10	(E)-2,3-bis(7-methoxy-2-oxochromen-8-yl)prop-2-enal	10597338	C_23_H_16_O_7_

### PPI network and hub genes

**FIGURE 3:**
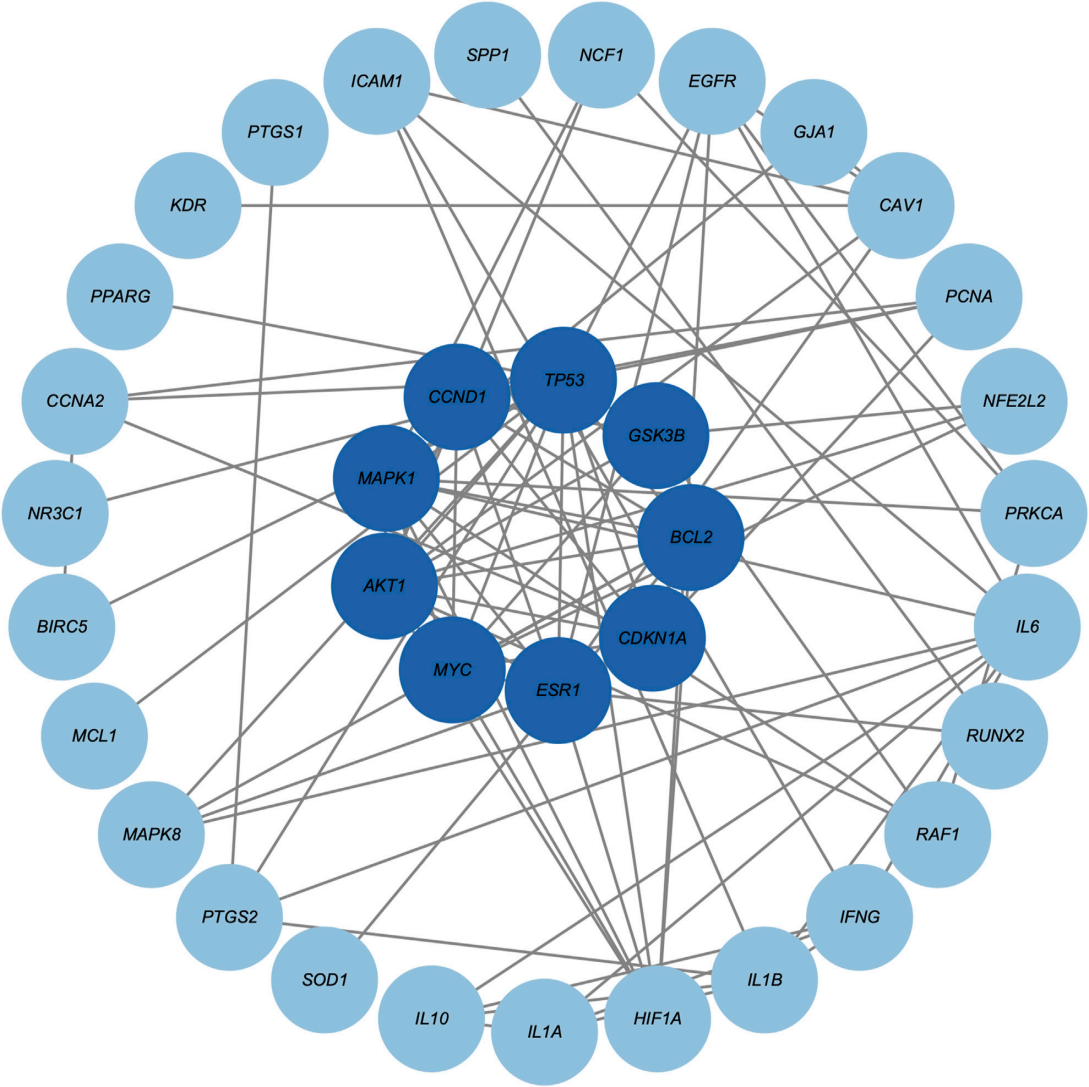
Protein-protein interaction network of core genes. Darker blue circle nodes in the inner circle indicate the nine screened hub genes.

Based on the 42 core genes, we constructed a PPI network containing 36 nodes and 93 edges (Figure 3). After one round of screening, nine hub genes were identified, namely, *AKT1*, *MAPK1*, *CCND1*, *TP53*, *GSK3B*, *BCL2*, *CDKN1A*, *ESR1*, and *MYC*. Supplementary Table S5 provides detailed scoring data used for hub gene screening.

### GO and KEGG enrichment analyses

**FIGURE 4:**
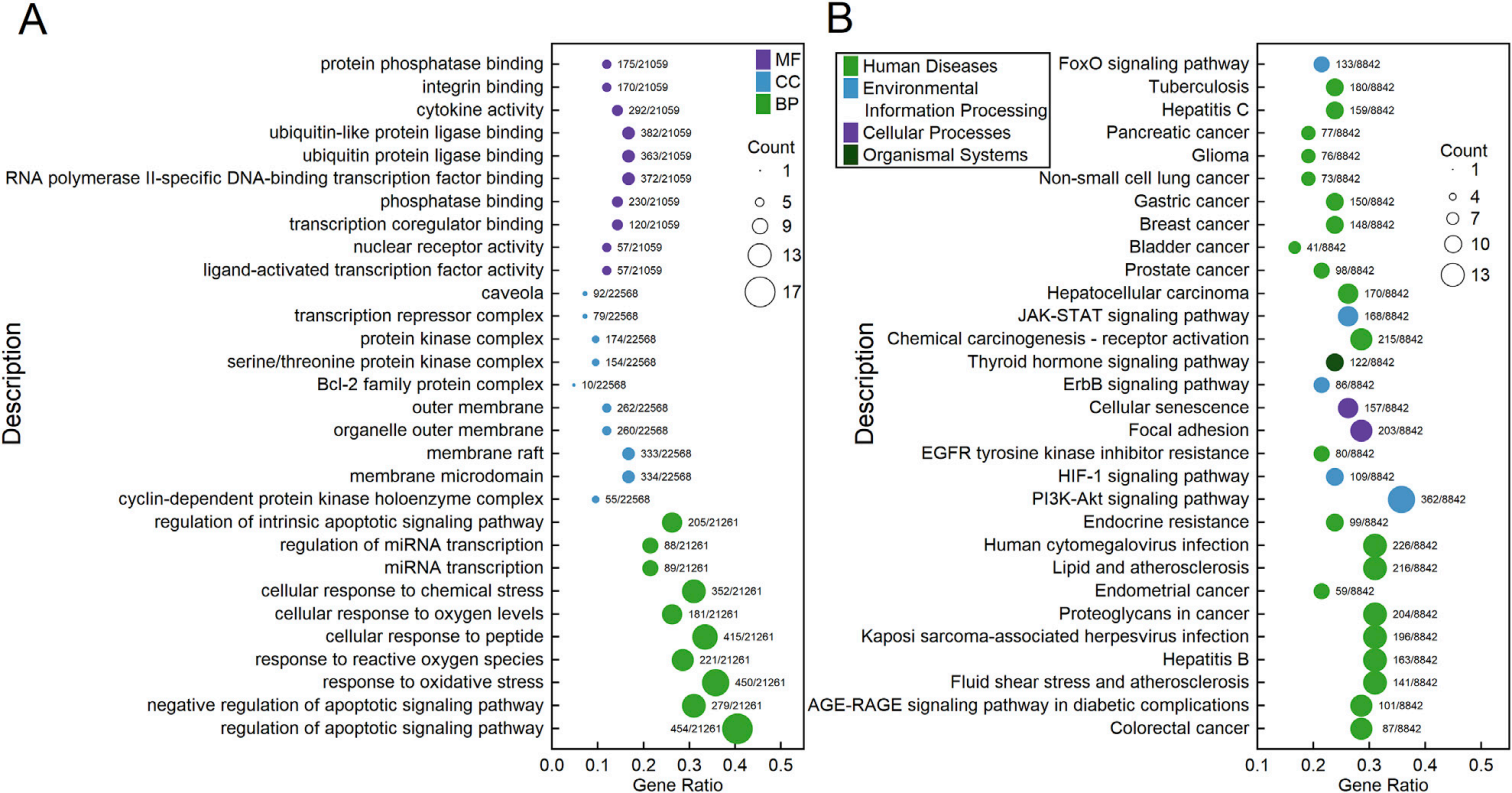
Bubble charts showing the results of GO **(A)** and KEGG **(B)** enrichment analyses of the 42 core genes. Each bubble represents a GO term **(A)** or a KEGG pathway **(B)**. The fill color of the bubbles indicates their categories, and the size of the bubbles indicates the count of genes enriched into that GO term **(A)** or KEGG pathway **(B)**. The “Gene Ratio” represents the ratio of the count of enriched genes to the total number of genes. The label alongside each bubble indicates the counts of genes in that GO term **(A)** or KEGG pathway **(B)** relative to the total number of genes in the category.

The 42 core target genes were significantly enriched (adjusted *p* < 0.05) into 1597 GO terms: 1842 biological process (BP) terms, 22 cellular component (CC) terms, and 93 molecular function (MF) terms. Figure 4A shows the top 10 most enriched BP, CC, and MF terms, including regulation of apoptotic signaling pathway, negative regulation of apoptotic signaling pathway, response to oxidative stress, response to reactive oxygen species, cellular response to peptide, cellular response to oxygen levels, cellular response to chemical stress, miRNA transcription, regulation of miRNA transcription, regulation of intrinsic apoptotic signaling pathway, et al. Supplementary Table S6 provides detailed information about these significantly enriched GO terms. KEGG enrichment analysis revealed 219 significantly enriched (adjusted *p* < 0.05) KEGG pathways: 20 in Cellular Processes, 29 in Environmental Information Processing, six in Genetic Information Processing, 90 in Human Diseases, eight in Metabolism, and 65 in Organismal Systems. Figure 4B shows the 30 most enriched KEGG pathways, including Colorectal cancer, AGE-RAGE signaling pathway in diabetic complications, Fluid shear stress and atherosclerosis, Hepatitis B, Kaposi sarcoma-associated herpesvirus infection, Proteoglycans in cancer, Endometrial cancer, et al. Supplementary Table S7 provides detailed information about these significantly enriched KEGG pathways.

### Molecular docking

**FIGURE 5:**
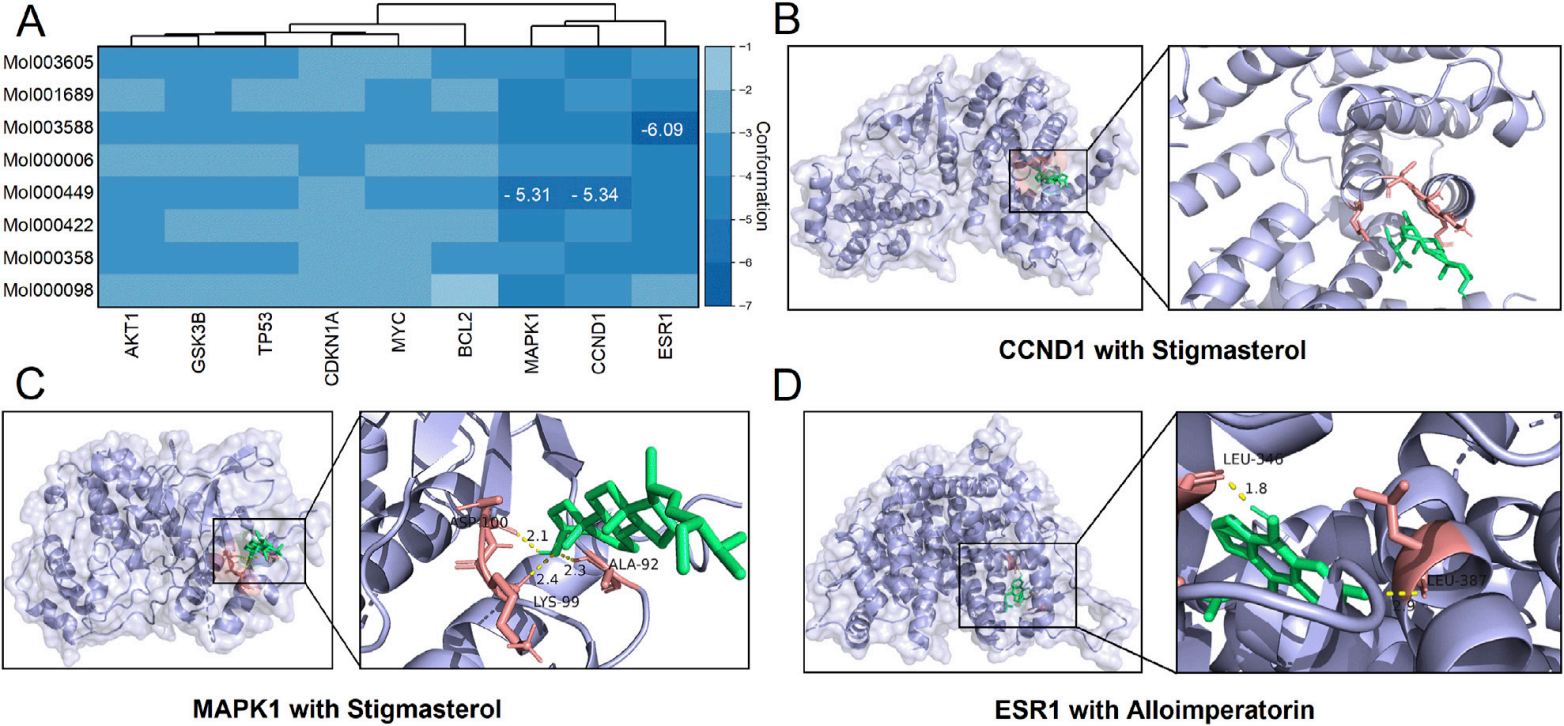
Results of molecular docking. **(A)** A clustered heatmap showing the affinities between each pair of docking conformations. **(B–D)** Molecular docking diagrams showing the conformation complexes of stigmasterol with Cyclin D1 (CCND1) and mitogen-activated protein kinase 1 (MAPK1), as well as the complex of alloimperatorin with estrogen receptor 1 (ESR1).

We docked proteins coded by these nine hub genes with the eight core active ingredients, generating a total of 72 conformation complexes (Figure 5A). The results revealed that three out of these 72 conformation complexes exhibited affinities lower than −5. Specifically, the Cyclin D1 (CCND1)-stigmasterol complex had an affinity of −5.34 and formed three hydrogen bonds at the ASP-100, ALA-92, and LYS-99 residues (Figure 5B). The mitogen-activated protein kinase 1 (MAPK1)-stigmasterol complex had an affinity of −5.31 but did not form hydrogen bonds (Figure 5C). Lastly, the estrogen receptor 1 (ESR1)-alloimperatorin complex had an affinity of −6.09 and formed two hydrogen bonds at the LEU-346 and LEU-387 residues (Figure 5D).

## Discussion

HSs are a condition with complex mechanisms that impose significant economic and psychological burdens on patients with wounds (Mony et al., 2023; Bharadia et al., 2023; Mcphail et al., 2022). Recently, various extracts from multiple TCM have been reported to show effects in preventing and treating HSs (Ning et al., 2021; Chen et al., 2022). SHO is a TCM prescription used for the external treatment of scarring, showing potential effects in treating HSs. However, the underlying mechanisms of SHO’s effects on HSs remain unexplored. In this study, using bioinformatic methods, we systematically explored the potential mechanisms of SHO in the treatment of HSs.

The SHO formula, developed from decades of clinical experience, has been used for external treatment of scaring since the 1960s, successfully treating thousands of patients with HSs. The SHO formula includes *Galla Chinensis*, *Fructus Mume*, *Radix Angelicae Dahuricae*, *Fructus Cnidii*, *Herba Senecionis Scandentis*, and *Cortex Lycii*. Based on the TCM theory of “qi and blood stasis, and toxic accumulation”, it follows the principle of “softening hardness, dissipating nodules, and detoxifying”. *Galla Chinensis*, the principal herb, has astringent, toxic-expelling, and hardness-softening properties, helping to reduce swelling and scar formation. Modern pharmacology shows it has powerful antioxidant properties, protecting biological macromolecules from damage (Ren et al., 2021). *Fructus Mume*, sour and astringent, complements *Galla Chinensis* by reducing scars (Zhang et al., 2023). *Radix Angelicae Dahuricae* promotes blood circulation, dissipates blood stasis, reduces swelling, and generates new tissue. *Fructus Cnidii* clears heat, dries dampness, dispels wind, and relieves itching. *Herba Senecionis Scandentis* clears heat, detoxifies, kills parasites, and relieves itching. Together, these herbs have antibacterial and anti-inflammatory effects, theoretically capable of controlling local inflammation, reducing pain and itching, and accelerating wound healing, thereby shortening the treatment duration.

We first retrieved the active ingredients in the SHO formula from the TCMSP database, and HSs-related target genes from five databases. Since there was no information about *Senecio Scandens* in the TCMSP database, we obtained its active ingredients from the HERB database. To ensure the rigor of our analyses, we cross-validated these active ingredients with the TCMSP database and used only those certified by the TCMSP database in our subsequent analyses. After a series of analyses, we identified 57 active ingredients and predicted 42 potential targets in the six herbs pf SHO (Table 1; Figure 1). Notably, since SHO is an external ointment, dermal permeability parameters should ideally be used as screening criteria. However, due to the limited number of network pharmacology studies focused on external drug applications, most databases do not provide dermal permeability parameters for active ingredients, and such data are generally unavailable. Consequently, this study employed pharmacokinetic parameters commonly used in other network pharmacology studies (Fan et al., 2024; Han et al., 2022) as the screening criteria. This limitation may affect the reliability of our findings and highlights the importance of incorporating dermal permeability parameters in future research. Network analyses indicated that SHO might affect HSs by regulating proteins such as AKT1, MAPK1, CCND1, TP53, GSK3B, BCL2, CDKN1A, ESR1, and MYC (Figure 3). GO and KEFF enrichment analyses showed that these targets are involved in multiple categories and pathways, such as the cytokine activity, regulation of apoptotic signaling pathway, response to oxidative stress, response to a steroid hormone, et al. (Supplementary Table S6; Supplementary Table S7), all of which are considered mechanisms of HSs (Mony et al., 2023; Gurtner et al., 2008; Diegelmann and Evans, 2004). Considering that SHO is used for external treatment, it is likely to act by influencing the response of myofibroblasts to regulatory factors in the microenvironment, such as cytokines and reactive oxygen species.

To further investigate the underlying molecular mechanisms, we performed molecular docking analyses of these hub target proteins and the core active ingredients in the SHO formula (Figure 2; Table 2). Although we identified three complexes with high affinities (Figure 5A), one of them did not form hydrogen bonds (Figure 5B), indicating that this conformation is unstable. The remaining two conformations, the MAPK1-stigmasterol complex, and the ESR1-alloimperatorin complex, showed high affinities and formed hydrogen bonds (Figures 5C,D), suggesting they are the most likely underlying molecular mechanisms of SHO’s effects on HSs. It should be noted that our docking results only offer preliminary indications of potential interactions. To more accurately assess binding stability, further validation through molecular dynamics simulations and binding free energy calculations is required.

Indeed, inhibition of the TGF-β1-ERK/JNK pathway has previously been found to be involved in scar formation. For example, basic fibroblast growth factor stimulates the proliferation of human dermal fibroblasts via the ERK1/2 and JNK pathways (Makino et al., 2010), and loureirin B has been found to inhibit the formation of HSs by inhibiting the TGF-β1-ERK/JNK pathway (He et al., 2015). Additionally, ESR1 is also identified to play important roles in skin aging and wound healing process (Saville and Hardman, 2014; Smith, 2017). These findings suggest that the mechanisms by which SHOW treats HSs may involve the regulation of ERK2 and ESR1 proteins. However, it is important to note that molecular docking results alone do not provide definitive evidence of molecular interactions. To address this limitation, binding assays should be conducted in wet-lab experiments to confirm these interactions, and further validation of the underlying mechanisms is warranted through both *in vivo* and *in vitro* studies.

## Conclusion

In summary, this study analyzed the effective targets and action pathways of the SHO formula and preliminarily explored its molecular mechanisms in treating HSs using network pharmacology and molecular docking methods. Our findings revealed that the main active ingredients of SHO include quercetin, beta-sitosterol, kaempferol, stigmasterol, luteolin, alloimperatorin, acacetin, and (E)-2,3-bis(7-methoxy-2-oxochromen-8-yl)prop-2-enal. The hub target proteins identified were AKT1, MAPK1, CCND1, TP53, GSK3B, BCL2, CDKN1A, ESR1, and MYC. GO and KEGG enrichment analyses indicated that these hub target genes are involved in processes and pathways related to apoptosis and responses to oxidants. Molecular docking analysis identified the MAPK1-stigmasterol and ESR1-alloimperatorin complexes as having high affinities and forming hydrogen bonds, suggesting that the SHO formula may affect the MAPK1 and ESR1 proteins, thereby preventing and treating HSs. Our study will provide new drug and target options for the prevention and treatment of HSs, and offer theoretical support for further research and promotion of SHO. However, further *in vivo* and *in vitro* studies are warranted to validate these mechanisms.

## Data Availability

The original contributions presented in the study are included in the article/Supplementary Material, further inquiries can be directed to the corresponding author.
